# Genetic and morphological data demonstrate hybridization and backcrossing in a pair of salamanders at the far end of the speciation continuum

**DOI:** 10.1111/eva.13312

**Published:** 2021-11-09

**Authors:** Jan W. Arntzen, Robert Jehle, Ben Wielstra

**Affiliations:** ^1^ Naturalis Biodiversity Center Leiden The Netherlands; ^2^ Institute of Biology Leiden University Leiden The Netherlands; ^3^ School of Science, Engineering and Environment University of Salford Salford UK

**Keywords:** hybrid phenotypes, interspecific gene flow, introgressive hybridization, newt, *Triturus*

## Abstract

Deeply diverged yet hybridizing species provide a system to investigate the final stages of the speciation process. We study a hybridizing pair of salamander species—the morphologically and genetically drastically different newts *Triturus cristatus* and *T*. *marmoratus*—with a panel of 32 nuclear and mitochondrial genetic markers. Morphologically identified hybrids are mostly of the F_1_ generation and mothered by *T*. *cristatus*. The sex ratio of the F_1_ hybrid class is reciprocally skewed, with a preponderance of females in *T*. *cristatus*‐mothered hybrids and males in *T*. *marmoratus*‐mothered hybrids. This amounts to the Haldane effect operating in one direction of the cross. Deeper generation hybrids are occasionally produced, possibly including F_1_ hybrid × backcross hybrid offspring. Interspecific gene flow is low, yet skewed toward *T*. *cristatus*. This asymmetry may be caused by hybrid zone movement, with the superseding species being predisposed to introgression. The persisting gene flow between deeply differentiated species supports the notion that full genetic isolation may be selected against. Conversely, published morphological data suggest that introgressive hybridization is detrimental, with digital malformations occurring more frequently in the area of sympatry. Finally, to assist field identification, both within the area of natural range overlap and concerning anthropogenic introductions elsewhere, we document the phenotypical variation of two generations of hybrids compared with both parental species. We suggest that fluctuating range boundaries, ecological segregation, cytonuclear incompatibilities and hybrid breakdown through Bateson*–*Dobzhansky*–*Muller incompatibilities all contribute to species integrity, despite incomplete isolation during secondary contact.

## 1 INTRODUCTION

It has long been realized that new genetic variants introduced to populations through gene flow can improve a population's response to natural selection (Fisher, 1930; Fitzpatrick & Reid, 2019). Similarly, introgressive hybridization leading to gene flow among species may be advantageous, because it allows an evolutionary lineage to “sample” genetic variants that have already undergone natural selection in another lineage, facilitating the spread of selectively beneficial alleles between otherwise reproductively isolated genetic backgrounds (Barton, 2020; Fraïsse et al., 2014; Seehausen et al., 2014). One example concerns the exchange of MHC genes among hybridizing species of *Lissotriton* newts (Nadachowska‐Brzyska et al., 2012). Hybridization may be considered an important evolutionary force that does not simply homogenize but may also recombine genetic variation (Abbott et al., 2013), such that genetic mixing has been proposed as a conservation tool in species reintroductions (Zecherle et al., 2021).

Speciation constitutes the achievement of reproductive isolation through the gradual build‐up of genetic and behavioral incompatibilities (Coyne & Orr, 2004). Genetic divergence requires genetic isolation, which is generally initiated in allopatry (Mayr, 1963). When diverging gene pools obtain secondary contact, the strength of reproductive barriers that have arisen can be put to the test. The outcome will be positioned somewhere at the continuum from a full merger to the complete genetic isolation of the differentiated gene pools (Stankowski & Ravinet, 2021; Wu & Ting, 2004). A regular observation for related species that hybridize upon secondary contact is that they geographically exclude one another, either with a narrow clinal hybrid zone forming their abutting range borders, or in a mosaic hybrid zone in which taxa map onto patches of interdigitated habitat (Barton, 1979; Harrison, 1986; Jiggins & Mallet, 2000). In particular, genetically deeply diverged yet hybridizing species provide a system to study the final stages of the speciation process. When hybridizing species have also diverged ecologically to the extent that interspecific competition is reduced, hybrid individuals—while rare—may be found scattered across the area of range overlap (Barton & Hewitt, 1985; Key, 1981).

**FIGURE 1:**
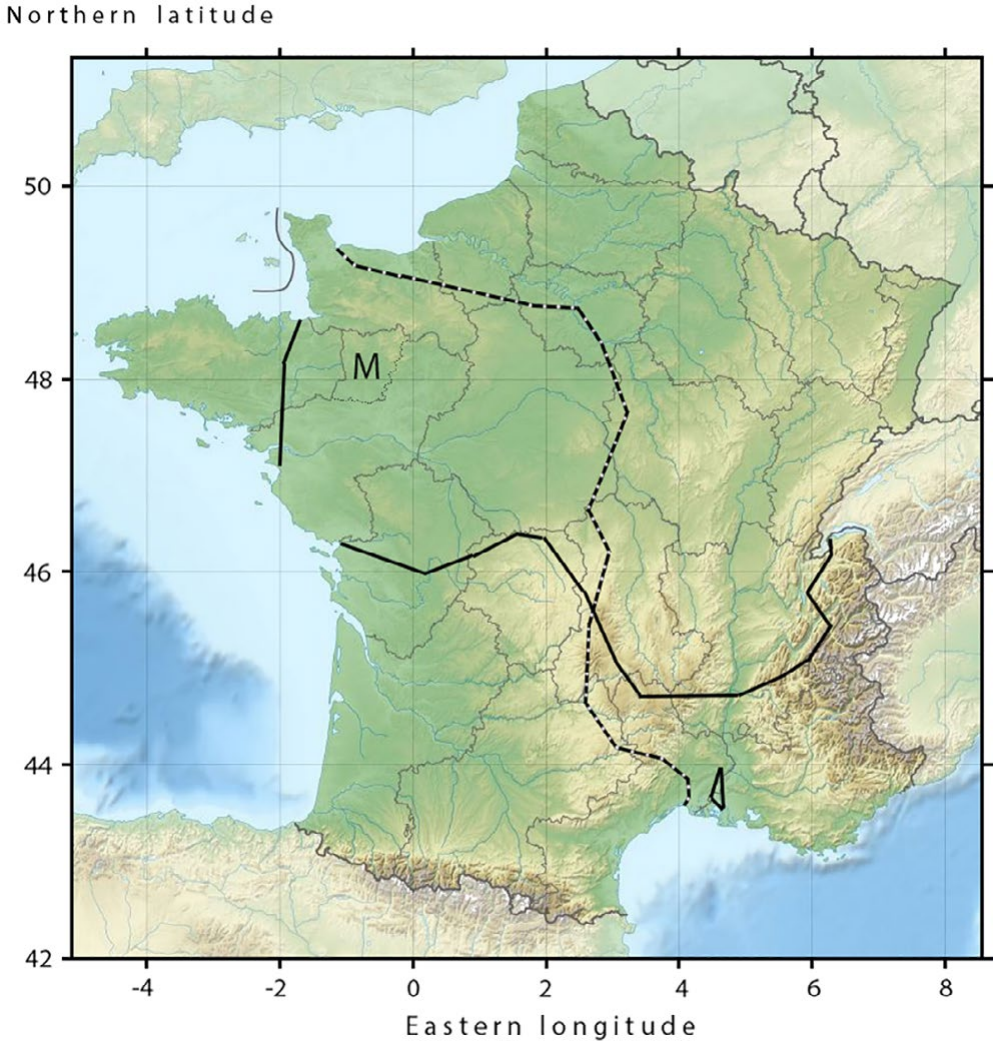
The southern range border of *Triturus cristatus* (solid line) and the northern range border of *T*. *marmoratus* in continental France (interrupted line) summarized from published data (Lescure & De Massary, 2012). M—department Mayenne. The base map was downloaded from https://www.mapsland.com

One example of a hybridizing pair of species that represents the “far side of the speciation continuum” (Hendry et al., 2009; Shaw & Mullen, 2014) is that of the crested newt *Triturus cristatus* (Laurenti, 1768) and the marbled newt *T*. *marmoratus* (Latreille, 1800). These species are distinct from the perspective of morphology and genetics, and last shared a common ancestor ca. 24 million years ago (Steinfartz et al., 2007). Range expansions after the last glacial maximum brought the two *Triturus* species back into contact across a currently ca. 300‐km‐wide zone of range overlap in central France (Lescure & de Massary, 2012; Figure 1). Within this zone, crested and marbled newts meet one another in ponds and other stagnant water bodies during the breeding season, where they hybridize. Field surveys revealed a substantial level of ecological segregation, with *T*. *cristatus* predominant in flat and open terrain and *T*. *marmoratus* occupying hilly and forested areas (Schoorl & Zuiderwijk, 1981; Visser et al., 2017). Hybrids make up only ca. 4% of the total adult population, so that the *T*. *cristatus–T*. *marmoratus* hybrid zone is distinctly bimodal (Arntzen & Wallis, 1991; Vallée, 1959).

We here study a large collection of morphologically identified *T*. *cristatus* ×* T*. *marmoratus* hybrids with a panel of two mitochondrial and 30 nuclear genetic markers, to test hypotheses raised but not fully answered in previous studies on this system as follows: (i) Most hybrids are F_1_ hybrids, that is, the direct offspring of *T*. *cristatus* and *T*. *marmoratus*, (ii) backcross hybrids are occasionally being formed, leading to interspecific gene flow, (iii) most F_1_ hybrids have a *T*. *cristatus* as their mother, and (iv) the sex ratio of the F_1_ hybrid class is reciprocally skewed, with a preponderance of females in *T*. *cristatus*‐mothered hybrids and males in *T*. *marmoratus*‐mothered hybrids. Furthermore, we document the phenotypes of various hybrid classes in comparison with those of both parental species, from photographs of the ventral sides of adults.

## 2 MATERIALS AND METHODS

### 2.1 Sampling and recording

Tail tips for genetic analysis were collected from newts at breeding sites in the département (department) Mayenne in the west of France, which is situated within the area of *T*. *cristatus* and *T*. *marmoratus* range overlap (Figure 1). All sampled individuals were adults that were identified from external morphology and coloration characters as either *T*. *cristatus* (*N* = 26), *T*. *marmoratus* (*N* = 61), or *T*. *cristatus* ×* T*. *marmoratus* hybrids (*N* = 123), with the exception of four larvae that were identified *a posteriori* from genetic data. A subset of the material was photographed at the ventral side of the body, where species’ coloration characteristics are most distinctive, namely orange with black dots in *T*. *cristatus* and dark gray with white stipples in *T*. *marmoratus*. We purposely focused on collecting morphologically intermediate individuals, including those that raised the suspicion of possibly being second‐generation hybrids (i.e., individuals with one or both parents that themselves are hybrids), or that otherwise seemed different from regular phenotypes (see photographic documentation below).

### 2.2 SNP discovery and validation

For nuclear DNA SNP marker design, we used sequence data for a panel of nuclear DNA markers produced using an Ion Torrent next‐generation sequencing protocol (Wielstra et al., 2014). In brief, we amplified markers of ca. 140 bp in length (excluding primers), positioned in 3′‐untranslated regions of protein‐coding genes, in five multiplex PCRs. We pooled the multiplexes for each individual and ligated unique tags to be able to recognize the product belonging to each individual. Upon amplicon sequencing, the output was processed with a bioinformatics pipeline that filters out poor‐quality reads and converts the data to consensus sequences in FASTA format. Diagnosticity was previously tested for one *T*. *cristatus* (UK) and two *T*. *marmoratus* (Spain) populations far outside the sympatric zone (Arntzen et al., 2021). Of the 52 nuclear DNA markers, *T*. *cristatus* and *T*. *marmoratus* had species diagnostic allele variants for 41 of them. We added one nuclear DNA marker from the literature (Espregueira Themudo et al., 2009). Mitochondrial DNA (mtDNA) was studied through species diagnostic SNPs at COI and ND4 (Visser et al., 2017; Wielstra et al., 2013).

For all markers, we determined informative SNPs by checking the sequence alignments by eye in MacClade 4.08 (Maddison & Maddison, 2005) and by using the Kraken software (LGC Genomics) to design primers for the SNP validation step. Genotyping was conducted at the SNP genotyping facility of the Institute of Biology, Leiden University, using the Kompetitive Allele‐Specific PCR (KASP) genotyping system (LGC Genomics). The KASP technology encompasses fluorescence‐based genotyping (Semagn et al., 2014). The SNP variant present in each individual (both variants in the case of a heterozygote) was determined in uniplex assays, based on two allele‐specific primers with a final base complementary to one of the two potential SNP variants that also possess a unique tail sequence. Different fluorescently labeled primers present in the KASP master mix correspond to each tail sequence and are activated when incorporated during subsequent PCR cycles, with further cycling causing signal intensity to increase. Considering the vast genome of *Triturus* newts (Gregory, 2020), and because the nuclear loci were chosen at random, they were assumed to be unlinked. Analyses were performed on the assumption that triploid individuals were absent or rare (Borkin et al., 1996). Two loci that showed a lack of heterozygotes were excluded, as were ten loci for which more than half of the data was missing. Altogether, there were 259 missing data points over 30 nuclear loci and 210 individuals (4.1%). Alien alleles are defined as those found in *T*. *marmoratus* but that are diagnostic for *T*. *cristatus*, and *vice versa*. Transgressing alleles are those found homozygous in hybrids.

### 2.3 Classification of observed and simulated hybrids

Based on the nuclear DNA markers, all individuals were classified according to their hybrid index (HI) and observed heterozygosity (H_o_), adjusted for missing observations. Values for HI ran from zero, if only alleles typical for *T*. *cristatus* were found, through HI = 30 for F_1_ hybrids, to HI = 60 for pure *T*. *marmoratus*. Observed heterozygosity ran from zero if no heterozygotes were found to 30 when all nuclear loci were heterozygous. We also used a Bayesian approach implemented in the program NewHybrids (Anderson & Thompson, 2002) to assign individuals to user‐defined pure or hybrid classes. There are three possible classes for the first generation, six possible classes for the second generation, and 21 for the third generation, of which 15 can potentially be discriminated. All entirely homozygous and heterozygous individuals were tagged as “class known” with the program's “z‐option,” as either *T*. *cristatus* (z0), *T*. *marmoratus* (z1), or F_1_ hybrid (z2). We ran the analysis with a burn‐in of 100,000 followed by 100,000 sweeps under default parameter settings, with genealogical depths of two or three generations. The same analytical procedure (but without the z‐option) was applied to a virtual population of 1000 *T*. *cristatus*, 1000 *T*. *marmoratus*, 1000 F_1_ hybrids, and 1000 backcross hybrids in either direction (1000 *T*. *cristatus* × F_1_ hybrids and 1000 *T*. *marmoratus* × F_1_ hybrids) that was created in silico with HybridLab (Nielsen et al., 2006), with the empirical data as a template. Finally, mtDNA SNPs were identified as representing “cristatus” or “marmoratus” haplotypes, for 208 out of 210 individuals (missing data 1.0%).

## 3 RESULTS

**FIGURE 2:**
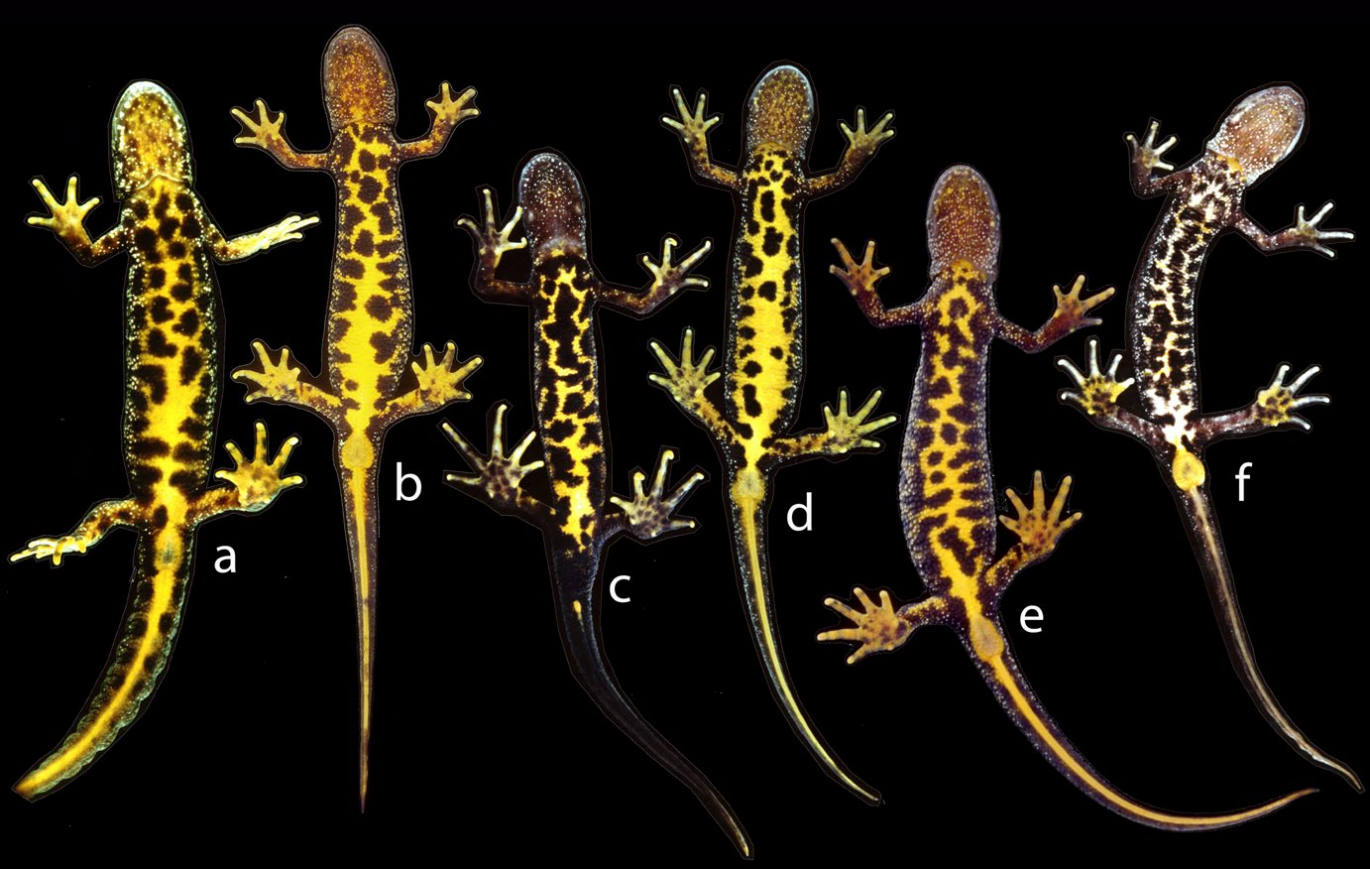
Six adult crested newts, *Triturus cristatus*, photographed at their ventral side. All are females except for c. In this male, note the large, black cloaca, the long fingers and toes, and the black underside of the tail, except for a yellow spot just behind the cloaca. Individual f has an aberrant appearance with the yellow color not well developed, but genetic data did not reveal *T*. *marmoratus* genes in this or other specimens shown (Arntzen & Wallis, 1991, and present paper). Note that the black ventral dots of *T*. *cristatus* tend to increase in number and size with age so that an almost entirely black belly is not necessarily a feature derived from *T*. *marmoratus* (Arntzen & Teunis, 1993; Hinneberg et al., 2020). Whether this phenomenon applies to hybrids is not known, but the dark pattern observed in some individuals (such as individuals o, p, v, w and y in Figure 4) would suggest it does, as perhaps might be expected because hybrids show high longevity (Cogălniceanu et al., 2020; Francillon‐Vieillot et al., 1990)

**FIGURE 3:**
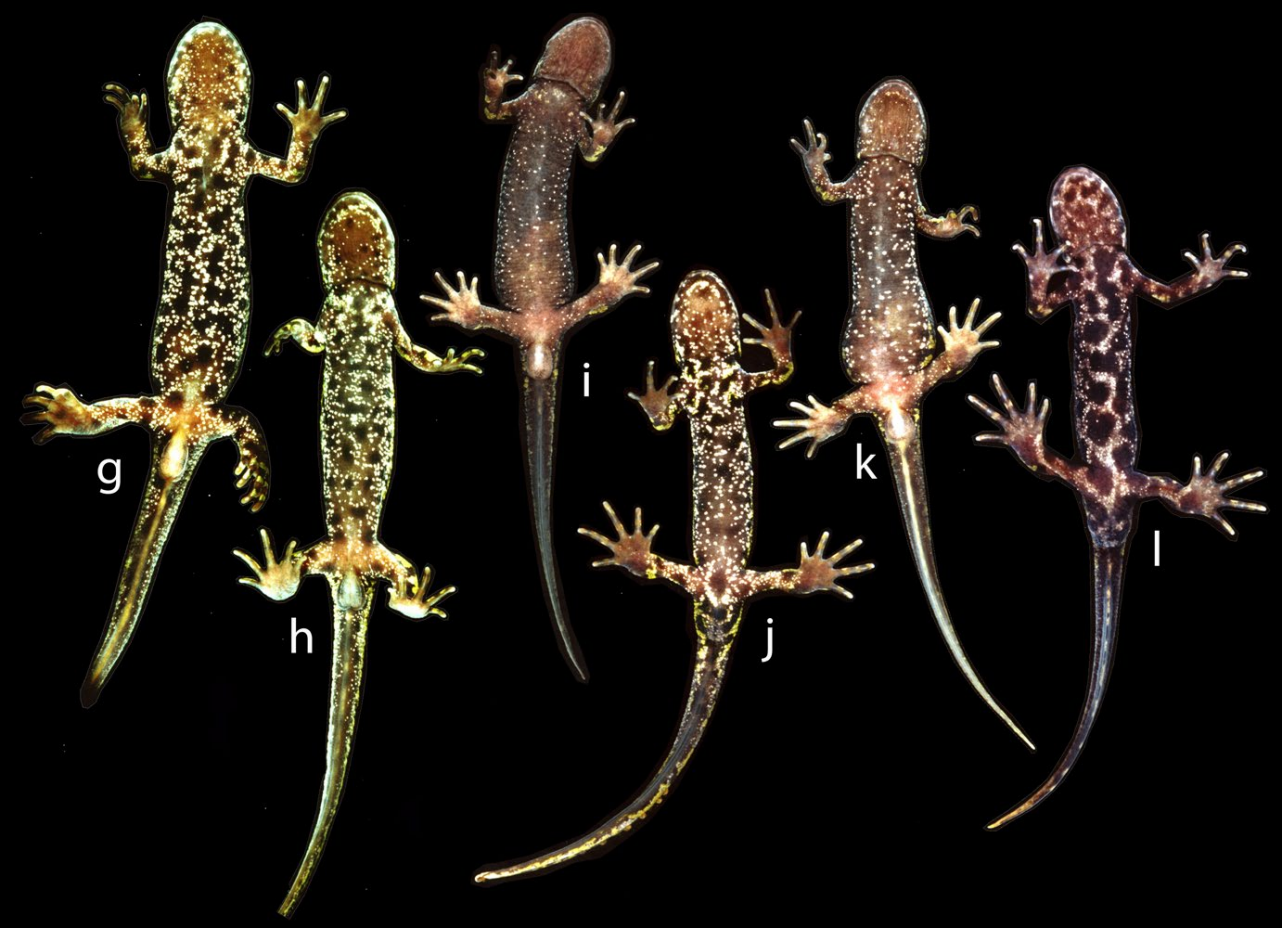
Six adult marbled newts, *Triturus marmoratus*. All are females except for individuals j and l. In these males, note the large and dark cloaca and the long fingers and toes. Individual l shows a pattern of irregular, sometimes interconnected large black dots that is also seen in *T*. *cristatus*. However, genetic data did not reveal *T*. *cristatus* genes in this or other specimens shown (Arntzen & Wallis, 1991, and present data)

**FIGURE 4:**
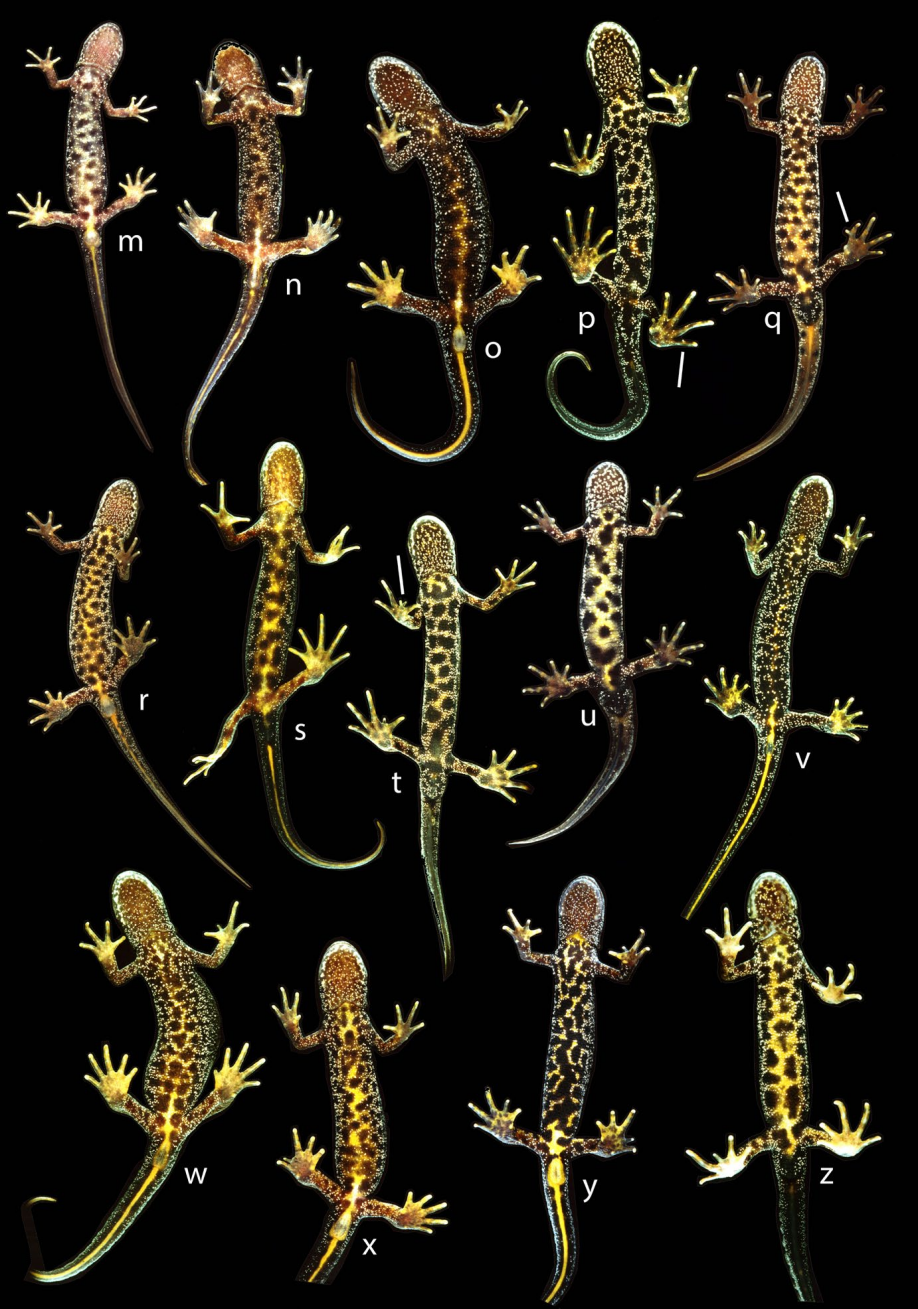
Fourteen *Triturus cristatus* × *T*. *marmoratus* hybrids. Individuals p, q, s, t, u and z are males, and the others are females. All individuals were subject to SNP analysis and classified as first‐generation hybrids. Individuals p and q have forked toes, and t has a forked finger, highlighted by white bars. Note that several fingers and toes were clipped for individual identification purposes. Some are mirror images for presentation purposes

**FIGURE 5:**
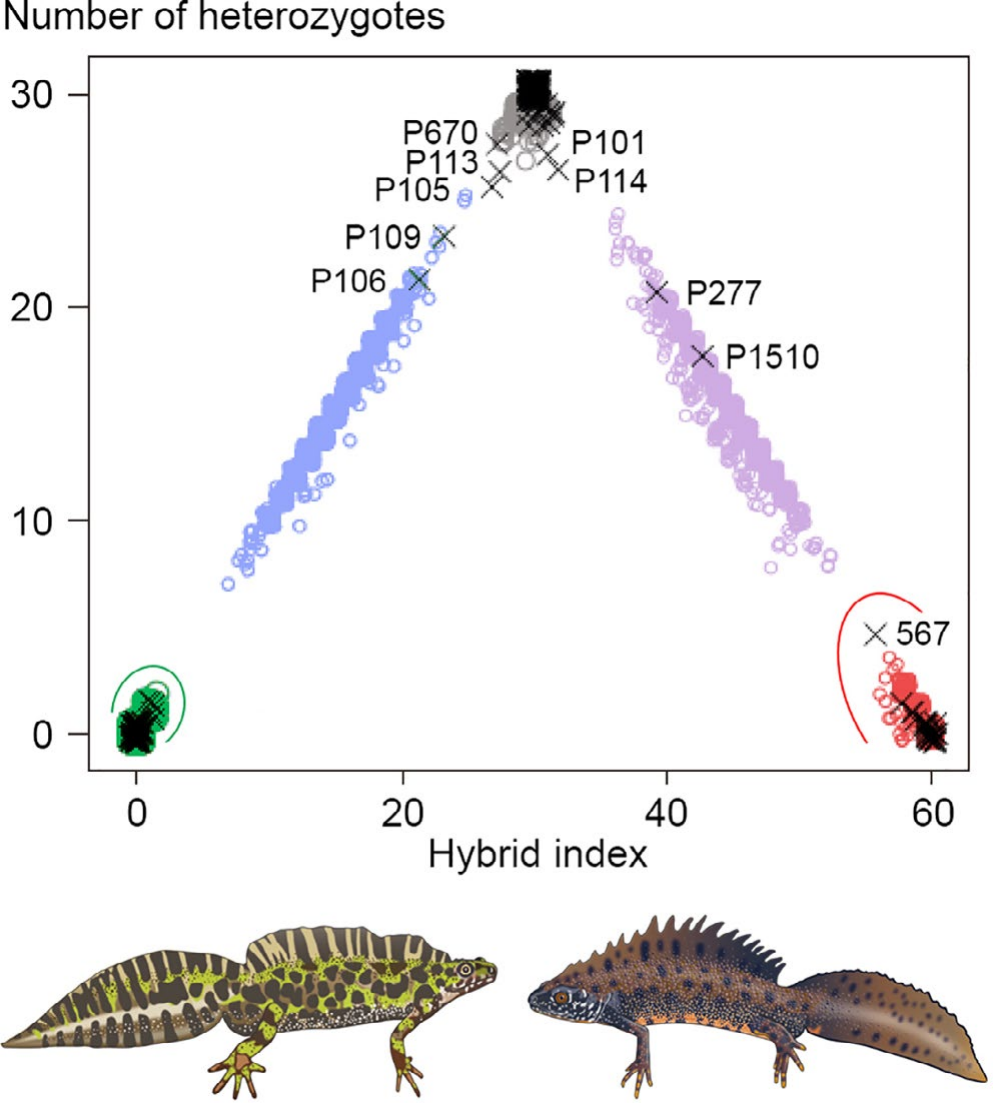
Classification of observed and simulated data on marbled newts (*Triturus marmoratus*, lower left corner), crested newts (*T*. *cristatus*, lower right corner), and their interspecific hybrids. First‐generation hybrids are in the top corner, and backcross hybrids are along the sides of the triangle. Observed data are shown by black crosses, and simulated data are shown by open round‐colored symbols (gray for F_1_ hybrids, blue and purple for backcross hybrids). A small amount of jitter was applied to reduce the overlap of equivalent data points. Adult newts with a position within the colored ellipses are phenotypically unblended *T*. *marmoratus* (green) and *T*. *cristatus* (red). Drawings at the bottom are by Bas Blankevoort © Naturalis Biodiversity Center

The field classification of adult newts in three morphological classes was mostly unproblematic, as is illustrated by the ventral side coloration characteristics of *T*. *cristatus* (Figure 2), *T*. *marmoratus* (Figure 3), and hybrids (Figure 4). A plot of HI versus H_o_ in the layout of an isosceles triangle has the parental species in the opposite lower corners and hybrids positioned at the top (Figure 5). Under reference to the simulated data, the vast majority of individuals that are not *T*. *cristatus* or *T*. *marmoratus* is attributed to the F_1_ hybrid category (118 or 119 out of 123, ~96%), including the individuals shown in Figure 4. The spread of the observed data points in Figure 5 is wider than is accounted for by the simulated data. This applies to individuals P101 and P670, which classify as F_1_ hybrids, and individual 567, which shows and classifies as *T*. *cristatus*. Four individuals classify as backcross hybrids, two in the direction of *T*. *cristatus* (individuals P277 and P1510) and two in the direction of *T*. *marmoratus* (P106 and P109). Individuals P113 and P114 are probably F_1_ hybrids, and the status of P105 is ambiguous. The NewHybrids classifications under a genealogical depth of two generations support these interpretations with high probabilities (*p*_NH_ > 0.97), including individual 567 (*p*_NH_ = 0.98). Individual P105 classifies as an F_1_ hybrid (Table 1). Under a considered depth of three generations, results are the same for five individuals (P101, P113, P114, P670, and P1510), albeit with on average lower probability values (~*p*_NH_ = 0.85, range 0.73–0.99). Four individuals are classified as the offspring of an F_1_ hybrid and a backcross hybrid, in which the grandparental species is either *T*. *cristatus* (individual P277) or *T*. *marmoratus* (individuals P105, P106, and P109). Finally, with three alleles diagnostic for *T*. *marmoratus*, individual 567 may be a backcross hybrid (*p*_NH_ = 0.70) or a *T*. *cristatus* (*p*_NH_ = 0.30; Table 1).

**TABLE 1:** Classification of *Triturus* newts from their SNP genetic profile with NewHybrids, for individuals for which the outcome depends on the genealogical depth considered of two (left panel) or three (right panel) generations, at *p*_NH_‐values >0.05

Individual	Two generations		Three generations			
			Top tier		Runner up	
	Category	*p*_NH_	Category	*p*_NH_	Category	*p*_NH_
P101	F_1_	1.00	F_1_	0.73	(c*F_1_)*F_1_	0.25
P105	F_1_	1.00	(m*F_1_)*F_1_	0.58	F_1_	0.27
P106	m*F_1_	0.97	(m*F_1_)*F_1_	0.95		
P109	m*F_1_	1.00	(m*F_1_)*F_1_	0.96		
P113	F_1_	1.00	F_1_	0.81	(m*F_1_)*F_1_	0.13
P114	F_1_	1.00	F_1_	0.99		
P277	c*F_1_	1.00	(c*F_1_)*F_1_	0.86	c*F_1_	0.13
P670	F_1_	1.00	F_1_	0.95		
P1510	c*F_1_	1.00	c*F_1_	0.75	(c*F_1_)*F_1_	0.23
567	c	0.99	c*(c*F_1_)	0.70	c	0.30

Note

that these are the same individuals that are highlighted in Figure 1. For the constituting data, see Supporting Information. Codes applied are as follows: c—*T*. *cristatus*, m—*T*. *marmoratus*, and F_1_—*T*. *cristatus* ×* T*. *marmoratus*.

Across the 30 nuclear markers, eight alien alleles were found over 26 otherwise pure *T*. *cristatus* (1.07%) and five over 61 otherwise pure *T*. *marmoratus* (0.28%), a frequency distribution that is statistically significant from random expectations (G test for independence, G = 5.72, *df* = 1, *p *< 0.05). At the individual level, five *T*. *cristatus* (19.2%) and five *T*. *marmoratus* (8.2%) had one or more alien alleles, an imbalance that is not statistically significant (G = 2.02, *df* = 1, *p *> 0.05). In F_1_ hybrids, the frequency of transgressing alleles (0.59%; 11.9% at the level of individuals) was intermediate to that observed for alien alleles in the parental species. Transgressing *T*. *cristatus* alleles were four times more frequent than transgressing *T*. *marmoratus* alleles, which is different from the 1/1 expectation (G test for goodness of fit, G = 7.20, *df* = 1, *p *< 0.01). An imbalance in the opposite direction was noted for morphology, where two *T*. *cristatus* (7.7%) and 17 *T*. *marmoratus* (27.9%) were field‐annotated as “possibly with some influences of the other species.”

All *T*. *cristatus* and *T*. *marmoratus* carried the mtDNA haplotype that is typical for their nuclear genetic species identity. Among hybrids, 100 had the cristatus haplotype and 21 the marmoratus haplotype, which is significantly different from a 1/1 ratio (G test for goodness of fit, G = 51.6, *df* = 1, *p *< 0.0001). Among F_1_ hybrids with the cristatus or marmoratus haplotype, 34 and 17 were male, and 61 and three were female, respectively. Accordingly, the sex ratio across mtDNA‐defined groups was significantly biased (G test for independence, G = 17.1, *df* = 1, *p *< 0.0001). Analyzed per mtDNA class, the results are also significant (G test for goodness of fit; cristatus mtDNA G =7.67, *df* = 1, *p *< 0.01, marmoratus mtDNA G = 9.80, *df* = 1, *p *< 0.01) but with deviations in opposite directions (see male/female numbers above). Among the five possible backcross individuals, P105, P106, and P109 are adult males with the cristatus mtDNA haplotype, P1510 is a larva with the cristatus haplotype, and P277 is an adult male with the marmoratus haplotype.

The illustrated *T*. *cristatus* individuals all show the regular crested newt phenotype, with a uniform yellow to orange belly furnished with black dots (Figure 2). Individual f, while aberrant by its light‐yellow belly color, shows no obvious *T*. *marmoratus* morphological features, and marbled newt alleles were not found. The illustrated *T*. *marmoratus* individuals (Figure 3) have a soot black (g and h) or gray belly (i‐l) with conglomerates of white stipples that range from frequent (g, h and j) to medium (k) and sparse (i and l). Large black spots range from vague (i and k) to pronounced (j and l). In some cases, black spots could be considered the by‐product of clusters of white stipples (g and h). Individual l has particularly large black spots reminiscent of those in *T*. *cristatus*, but crested newt alleles were not observed.

Fourteen field‐identified and genetically confirmed *Triturus cristatus* × *T*. *marmoratus* F_1_ hybrids are illustrated in Figure 4. All individuals possess coloration characteristics of both species, and their field identification as hybrids was unproblematic, even though morphological variation is substantial. In rare cases of doubt, such as individuals p and v, it helped to inspect the dark dorsal side for traces of green coloration, as would seem to be derived from *T*. *marmoratus*.

**FIGURE 6:**
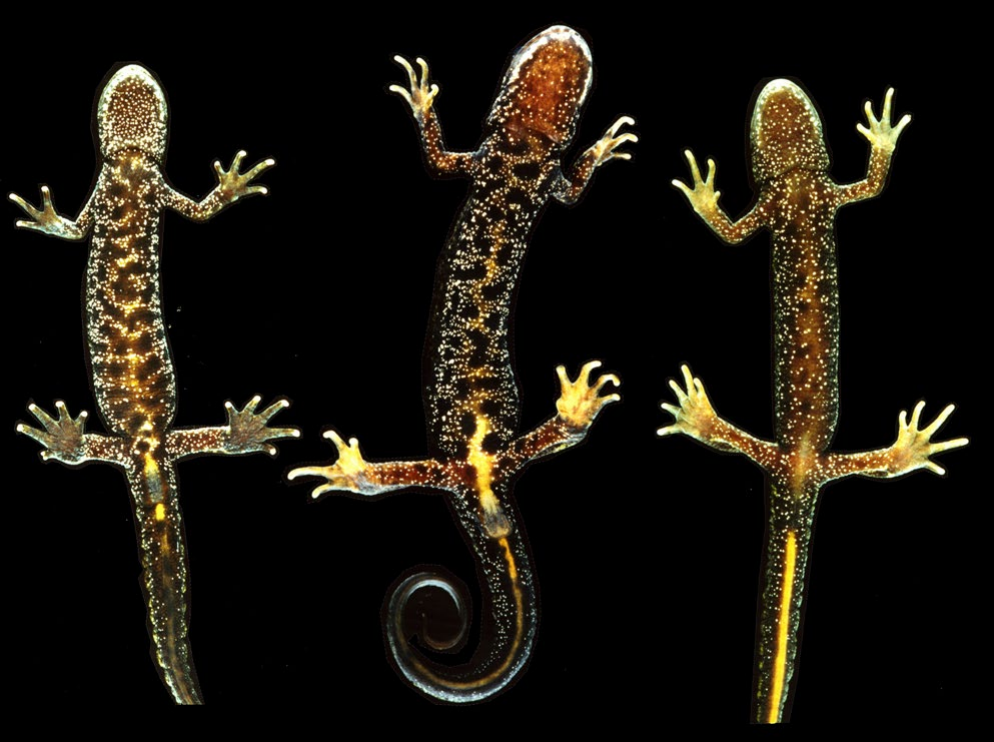
*Triturus cristatus *×* T*. *marmoratus* hybrids P105 (left), P109 (middle), and P106 (right). These three individuals are presumed to be backcrosses in the direction of *T*. *marmoratus* (F_1_ hybrid × *T*. *marmoratus*), but classification results with NewHybrids depend on the genealogical depth considered (for details, see text and Table 1). Note that backcrosses in the direction of *T*. *cristatus* were also found (individuals P1510 and P277), but these are without photographic documentation

Four or five individuals were genetically classified as backcross hybrids. No photographic documentation is available for the adult male P277 and the larva P1510. Individuals P106 and P109 were field‐classified as possible backcrosses with *T*. *marmoratus* on the basis of a predominantly black belly with white stipples and traces of orange in the middle (Figure 6). Individual P105 was field‐classified as an F_1_ hybrid, whereas the genetic data support either an F_1_ hybrid or backcross hybrid identity, depending on the analysis (Table 1).

## 4 DISCUSSION

The speciation continuum can be defined as the continuous sequence of genetically based changes that occur as two lineages diverge from one another on the pathway to reproductive isolation. Under the classic view of allopatric speciation developed in the 19^th^ and early 20^th^ century, reproductive isolation arises and reaches completion in the absence of gene flow and is maintained during subsequent secondary contact (Mayr, 1963). This model remains widely accepted as a basic scenario for the origin of biodiversity, but was also followed by the recognition of a broader range of mechanisms that possibly lead to species and their properties (Coyne & Orr, 2004). For example, we now know that taxa can diverge in the face of limited gene exchange when exposed to gradual environmental clines in parapatric conditions (Pinho & Hey, 2010; Roux et al., 2016; Shafer & Wolf, 2013). Genomic inferences further revealed that incomplete isolation between species is widespread and that “ancient” admixture during species’ evolutionary histories can be a driving force for radiations (for a review, see Taylor & Larson, 2019). However, genomic signatures of past gene flow do not necessarily reflect current rates of hybridization (Barth et al., 2020; Hime et al., 2021; Rancilhac et al., 2021) and only the latter is relevant for proximate mechanisms that complete the process of speciation.

Gene exchange between species is initiated by hybridization, which in nature is facilitated by incomplete reproductive barriers, followed by successful reproduction of F_1_ hybrids with either parental species (Mallet, 2005). Most of the hybrid newts we found were F_1_ hybrids, that is, the direct offspring of *T*. *cristatus* and *T*. *marmoratus*, but we also identified five backcross hybrids. These backcross hybrids represent independent observations because they represented different life stages, had different species as their nonhybrid (grant)parent, were sampled over a long time frame, and originated from different ponds. The depth of backcrossing could not always unequivocally be determined, either due to the limited resolution of the panel of nuclear markers and missing data or, possibly, genotyping errors and physical linkage. Given the low frequency of F_1_ individuals in the bimodal hybrid zone (ca. 4% of the total adult population; Arntzen & Wallis, 1991; Vallée, 1959), it was unexpected that four out of five second‐generation backcross individuals were classified by NewHybrids as descending from two successive matings involving an F_1_. For crosses between a first‐generation backcross and an F_1_, the species‐specific alleles are expected to be apportioned as 37.5% and 62.5% (combining 25%/75% and 50%/50%). This is broadly the case for individuals P106 (35% *T*. *cristatus* alleles, 65% *T*. *marmoratus* alleles), P109 (38% *T*. *cristatus* alleles, 62% *T*. *marmoratus* alleles) and P277 (65% *T*. *cristatus* alleles, 35% *T*. *marmoratus* alleles), but not for P105 (46% *T*. *cristatus* alleles, 54% *T*. *marmoratus* alleles; see Supporting Information for the SNP data). The latter individual also contains a SNP that is homozygous for the *T*. *cristatus* allele, which is not compatible with a (m*F_1_)*F_1_ genotype and suggests that it is better classified as F_1_. Whether backcrossing with an F_1_ might represent a fitness advantage in view of, for example, Bateson*–*Dobzhansky*–*Muller incompatibilities cannot be addressed at the given genetic resolution. Considering that backcrosses were preferentially sampled and nevertheless only formed 4.1% of the hybrid class (a class that itself is at ca. 4%), they appear to be exceedingly rare, presumably at <0.1% of the total *Triturus* population even under syntopic conditions. That the reproductive success of F_1_ hybrids in this system might be compromised was deduced early on from failures in spermatogenesis, reduced female fecundity, and embryo hatching success (Arntzen & Hedlund, 1990; Lantz, 1947; Lantz & Callan, 1954; Vallée, 1959; White, 1946). A low level of genetic leakage between *T*. *cristatus* and *T*. *marmoratus* is supported by the infrequent occurrence of alien alleles in morphologically unblended individuals and by malformations at fingers and toes that are attributed to introgressive hybridization (Table 2). These observations reinforce a strong role of postzygotic isolation for species cohesion at this level of diversification.

**TABLE 2:** Overview on genetic and morphological data in support of introgressive hybridization in *Triturus cristatus–T*. *marmoratus*

	*T. cristatus*		*T. marmoratus*		F_1_ hybrids		G test		
	Present	Absent	Present	Absent	Present	Absent	Type	Test value	Result
Alien alleles									
Genes	29	7711	24	9768			I	2.39	NS
Individuals	24	406	23	521			I	0.95	NS
Rare alleles									
Genes	71	7669	1	9791			I	107.1	***
Individuals	56	374	1	543			I	86.9	***
Digital malformations									
Individuals	32	669	36	535			I	1.87	NS
Individuals	Parental species pooled				11	54	I	10.5	**
	*T. cristatus*		*T. marmoratus*		F1 hybrids				
	Present	Absent	Present	Absent	*T. cristatus*	*T. marmoratus*			
Alien alleles									
Genes	8	741	5	1788			I	5.75	*
Individuals	5	21	5	56			I	2.02	NS
Transgressing alleles									
Genes					16	4	F	7.20	**
Individuals					13	2	F	8.07	**

Note

Top panel—published data on allozymes and digital malformations (Arntzen & Wallis, 1991). Rare alleles are here interpreted as “hybrizymes,” that is, genetic variants derived from hybridization. Bottom panel—SNP data present study.

I—G test of independence, F—G test of goodness of fit.

NS, not significant. *p < 0.05;**p < 0.01; and ***p < 0.001.

Another novel finding is that of adult F_1_ female hybrids that result from a male *T*. *cristatus* as a father and a *T*. *marmoratus* as the mother. The three individuals involved were found in two ponds, suggesting at least two independent mating events. This class of hybrids was not documented in earlier surveys, presumably because of smaller samples and their apparent rarity. The more common *T*. *cristatus*‐mothered hybrid class shows a shortage of the male heterogametic sex, suggesting a Haldane effect (Haldane, 1922; Schilthuizen et al., 2011). Conversely, the much rarer *T*. *marmoratus*‐mothered class is dominated by males, which can be explained by an incompatibility between the *T*. *cristatus* X chromosome and *T*. *marmoratus* cytoplasm, therewith overriding a Haldane effect, if it would operate (Arntzen et al., 2009).

The spread of alien alleles following hybridization is constrained by a breakdown due to Bateson*–*Dobzhansky*–*Muller genetic incompatibilities, the perseverance of which, however, depends on levels of underlying genetic drift and recombination (Xiong & Mallet, 2021). We observed an exceedingly low, yet asymmetric level of interspecific gene flow, therewith confirming the indications previously obtained with allozymes (Arntzen & Wallis, 1991). Asymmetric gene flow may be associated with, among others, a moving hybrid zone, for which introgression is expected to be more pronounced in the advancing than in the receding species (Barton & Hewitt, 1985; Currat et al., 2008; Quilodrán et al., 2020). Accordingly, the direction of the asymmetry points to *T*. *cristatus* as the invading species. This scenario is supported by historical data that document hybridization and incomplete species replacement, with *T*. *cristatus* taking over from *T*. *marmoratus* at a speed of ca. 1 km per year (Arntzen & Wallis, 1991).

Aside from demonstrating the occurrence of backcross individuals in natural populations, we also document their phenotypes for future reference. All four backcrossed adults were recognized as such in the field, including the correct identification of the nonhybrid (grand)parent. However, other candidates for a backcross status turned out to be F_1_ hybrids or (occasionally) pure *T*. *cristatus* or *T*. *marmoratus*. The latter observation suggested that variation in color pattern of either species was wider than anticipated, especially in *T*. *marmoratus*. The photographs of the ventral sides of two generations of genetically classified *T*. *cristatus–T*. *marmoratus* hybrids along with both parental species may assist identification in the field, either within the area of range overlap, or as derived from anthropogenic introductions such as in the United Kingdom (Arntzen et al., 2009) and the Netherlands (Beukema et al., 2015).

To conclude, *T*. *cristatus* and *T*. *marmoratus* form a well‐characterized zone of secondary contact where hybridization is infrequent. We employed a suite of diagnostic SNPs to candidate admixed individuals and confirmed the occurrence of concurrent bidirectional introgression. This finding reveals the consequential nature of hybridization between *T*. *cristatus* and *T*. *marmoratus*, and adds to the emerging general notion that complete reproductive isolation could be selected against (Barton, 2020). In our study system, however, interspecific gene flow, limited as it may be, seems to also come at a fitness cost. In hybrid individuals, we frequently observed polydactyly and other malformations at the fingers and toes (16.9%; Table 2, Figure 4), more so than in the parental species in sympatry (5.4%) and in allopatry (ca. 1.2%; Arntzen & Wallis, 1991; Vallée, 1959) and we occasionally observed severe cases such as a supernumerary arm or leg growing from the trunk (polymely; JWA, unpublished; see also Henle et al., 2017). To quantify the fitness cost of interspecific gene flow, it may be worthwhile to study the frequency and severity of malformations in embryos and larvae and their survival rates (Arntzen & Hedlund, 1990; Johnson et al., 1999). More broadly, studying systems at the far side of the speciation continuum enables us to identify the forces responsible for hybrid breakdown and strong reproductive isolation during the completion of speciation, which putatively differ from the drivers of diversification at initial phases of the speciation process (Kulmuni et al., 2020).

## CONFLICT OF INTEREST

The authors do not declare any conflict of interest.

## Supporting information

Supplementary Material

## Data Availability

The following data are already available from the Dryad digital repository (https://doi.org/10.5061/dryad.k0p2ngf63): raw Ion Torrent reads in FASTQ format, BWA alignments in SAM format, raw SNP reports in VCF format, filtered SNP report used to construct consensus sequences, overview of the number of reads in total and per marker and/or individual, and sequences in FASTA format, as developed and employed previously (Arntzen et al., 2021; Wielstra et al., 2014). The KASP‐data are available in FigShare under reference number https://doi.org/10.6084/m9.figshare.16783279.
